# Development and internal validation of machine learning algorithms for end-stage renal disease risk prediction model of people with type 2 diabetes mellitus and diabetic kidney disease

**DOI:** 10.1080/0886022X.2022.2056053

**Published:** 2022-04-04

**Authors:** Yutong Zou, Lijun Zhao, Junlin Zhang, Yiting Wang, Yucheng Wu, Honghong Ren, Tingli Wang, Rui Zhang, Jiali Wang, Yuancheng Zhao, Chunmei Qin, Huan Xu, Lin Li, Zhonglin Chai, Mark E. Cooper, Nanwei Tong, Fang Liu

**Affiliations:** a Division of Nephrology, West China Hospital of Sichuan University, Chengdu, China; b Laboratory of Diabetic Kidney Disease, Centre of Diabetes and Metabolism Research, West China Hospital of Sichuan University, Chengdu, China; c Division of Pathology, West China Hospital of Sichuan University, Chengdu, China; d Department of Diabetes, Central Clinical School, Monash University, Melbourne, Australia; e Division of Endocrinology, West China Hospital of Sichuan University, Chengdu, China

**Keywords:** Type 2 diabetes mellitus, diabetic kidney disease, end-stage renal disease, risk prediction model, machine learning

## Abstract

**Aims:**

Diabetic kidney disease (DKD) is the most common cause of end-stage renal disease (ESRD) and is associated with increased morbidity and mortality in patients with diabetes. Identification of risk factors involved in the progression of DKD to ESRD is expected to result in early detection and appropriate intervention and improve prognosis. Therefore, this study aimed to establish a risk prediction model for ESRD resulting from DKD in patients with type 2 diabetes mellitus (T2DM).

**Methods:**

Between January 2008 and July 2019, a total of 390 Chinese patients with T2DM and DKD confirmed by percutaneous renal biopsy were enrolled and followed up for at least 1 year. Four machine learning algorithms (gradient boosting machine, support vector machine, logistic regression, and random forest (RF)) were used to identify the critical clinical and pathological features and to build a risk prediction model for ESRD.

**Results:**

There were 158 renal outcome events (ESRD) (40.51%) during the 3-year median follow up. The RF algorithm showed the best performance at predicting progression to ESRD, showing the highest AUC (0.90) and ACC (82.65%). The RF algorithm identified five major factors: Cystatin-C, serum albumin (sAlb), hemoglobin (Hb), 24-hour urine urinary total protein, and estimated glomerular filtration rate. A nomogram according to the aforementioned five predictive factors was constructed to predict the incidence of ESRD.

**Conclusion:**

Machine learning algorithms can efficiently predict the incident ESRD in DKD participants. Compared with the previous models, the importance of sAlb and Hb were highlighted in the current model.

**Highlights**
- **What is already known?** Identification of risk factors for the progression of DKD to ESRD is expected to improve the prognosis by early detection and appropriate intervention.
- **What this study has found?** Machine learning algorithms were used to construct a risk prediction model of ESRD in patients with T2DM and DKD. The major predictive factors were found to be CysC, sAlb, Hb, eGFR, and UTP.
- **What are the implications of the study?** In contrast with the treatment of participants with early-phase T2DM with or without mild kidney damage, major emphasis should be placed on indicators of kidney function, nutrition, anemia, and proteinuria for participants with T2DM and advanced DKD to delay ESRD, rather than age, sex, and control of hypertension and glycemia.

## 1. Introduction

Diabetic kidney disease (DKD) is the most common cause of the end-stage renal disease (ESRD) [1]. Patients with ESRD must undergo dialysis or kidney transplantation to prolong survival, thus imposing a heavy burden on both the patients as well as the society. Early detection and improved management of people with type 2 diabetes mellitus (T2DM) and DKD may slow down the progression of DKD to ESRD, reduce its complications, and refine outcomes.

Recently, a number of studies have reported that factors, such as older age, sex, body mass index, control of glycemia, hypertension, dyslipidemia, proteinuria, and serum creatinine (sCr) level, appear to be traditional risk factors for the risk-scoring system for ESRD in patients with T2DM with or without mild kidney damage [2–4]. Although many risk factors involved in the progression of DKD, such as blood pressure, serum albumin (sAlb), serum uric acid (UA), severity of diabetic retinopathy (DR), and early-onset T2DM, have been reported [5–8], such a large number of indicators without relative importance can be difficult for clinicians to apply directly when making clinical decisions. Thus, it is necessary to construct a simple and applicable risk predictive model of ESRD to help clinicians identify the risk of their patients early and to provide early treatment to avoid progression to ESRD in patients with T2DM with DKD. Recently, Sun et al. [9] reported the construction of a predictive model for ESRD using logistic regression in Chinese patients with DKD confirmed by renal biopsy. However, the study had a relatively short follow-up period. Therefore, studies with a longer observation period and subjects from different cohorts are required to establish a more accurate predictive risk model for ESRD in patients with T2DM and DKD.

As a type of artificial intelligence in the field of computer science, machine learning uses statistical technique to give computers the capability to ‘learn’ particular assignments without being explicitly programmed. Multiple models of machine learning have been compared, and the random forest (RF) algorithm has been proven to have excellent performance, with high accuracy and superiority, and is usually better than logistic regression [10]. In the current study, we used machine learning algorithms to develop and validate an effective and accurate ESRD risk prediction model by using a larger sample of patients with DKD confirmed by renal biopsy. In addition, we developed an ESRD risk prediction nomogram for clinical application.

## 2. Subjects, materials and methods

### 2.1. Study population and study design

Three hundred and ninety Chinese patients with T2DM with DKD confirmed by percutaneous renal biopsy were enrolled between January 2008 and July 2019 and followed up for at least 1 year (with a median period of 3 years). The diagnosis of T2DM was made based on the criteria established by the American Diabetes Association [11]. The renal pathological classification of DKD was based on the Renal Pathology Society (RPS) 2010 criteria [12]. Enrolled participants met the following criteria: (1) e-GFR >15 mL·min^−1^ (1.73 m^2^)^−1^; and (2) ≥18 years old. Participants were excluded if they had started dialysis treatment before renal biopsy, had a history of renal transplantation, or completed less than 1 year of the follow-up period (Supplementary Figure 1). All participants signed a written informed consent form.

### 2.2. Laboratory and clinical characteristics

The following clinical characteristics were collected at baseline (renal biopsy): age, sex, 24-h urine urinary total protein (UTP), systolic/diastolic blood pressure (SBP/DBP), serum albumin (sAlb), blood urea nitrogen (BUN), UA, sCr, total cholesterol, triglyceride (TG), the presence of DR, high-density lipoprotein cholesterol (HDL-C), low-density lipoprotein cholesterol (LDL-C), glycosylated hemoglobin (HbA1c), hemoglobin (Hb), Cystatin-C (CysC), the eGFR (calculated with the equation of the CKD-EPI), the duration of T2DM, and the use of insulin, metformin, diuretics, CCB, ACEI, ARB, statins, and fibrates.

### 2.3. Renal biopsy-related information

Light microscopy, immunofluorescence, and electron microscopy were used to exam kidney biopsy specimens, and the pathological lesions were graded independently by two pathologists. The pathological classifications of glomerular alterations, interstitial inflammation, arteriolar hyalinosis, and interstitial fibrosis and tubular atrophy (IFTA) were on the basis of the criteria published by the Renal Pathology Society [12]. The deposition of C1q, IgG, IgM, IgA, C3, and C4 in kidney biopsy samples was also included in the analysis.

### 2.4. Outcome definition

The renal outcome was incident ESRD, defined as eGFR of <15 mL·min^−1^ (1.73 m^2^)^−1^, or requiring renal replacement therapy.

### 2.5. Statistical analysis

The multiple imputation method based on missForest was used to impute missing data [13]. Variables with more than 20% of missing data were excluded. Comparisons were made between the clinical, demographic, and pathological characteristics, and the treatment at biopsy between participants who had developed ESRD (ESRD group) and those who had not (control group) by logistic regression model. Continuous data were described using the mean and standard deviation and the categorical data were recorded as number and percentage. Significance was defined as a difference with a *p* <.05. R software version 4.0.2 was used to perform all statistical analyses.

### 2.6. Model development

**Figure 1.**
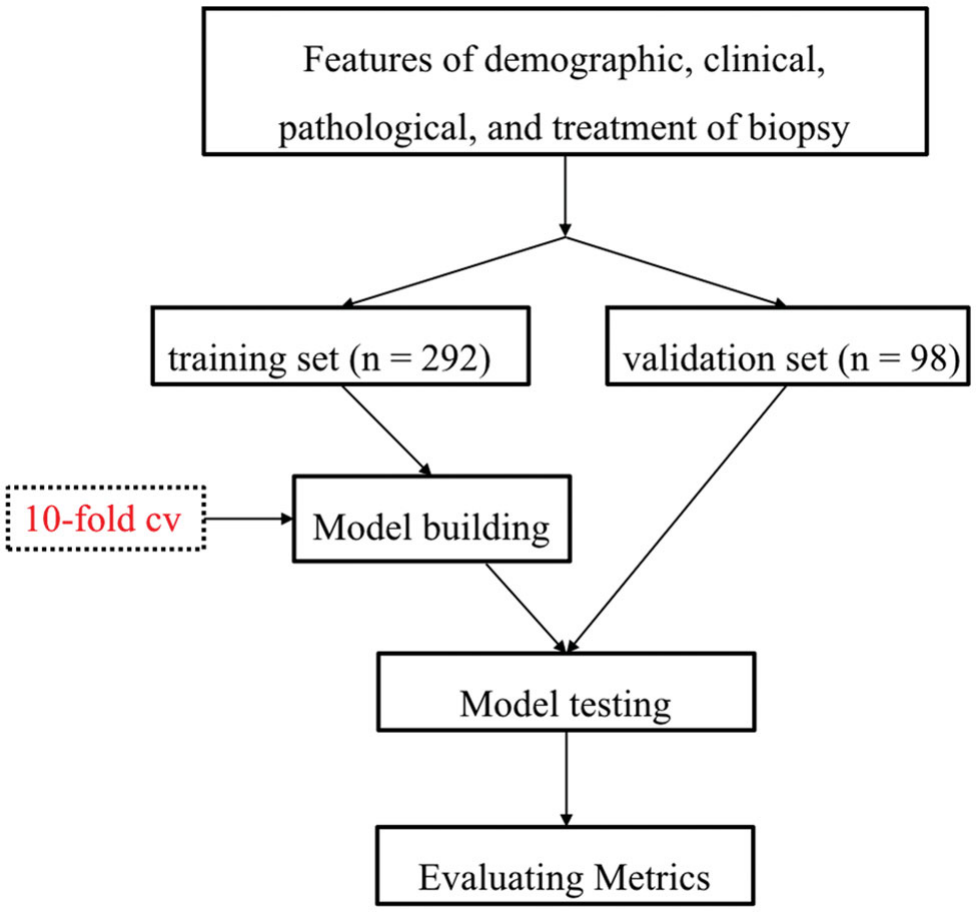
Process of establishing prediction models.

Four machine learning algorithms were utilized to select the critical features and build the risk prediction model: gradient boosting machine (GBM), support vector machine (SVM), RF, and logistic regression. The participants were randomly divided into training (75%) and validation (25%) sets. Models were developed using the Training Set (75% of data) and internally validated by the Validation Set (25% of data). A 10-fold cross-validation was made using the Training Set (75% of the full dataset), with one-tenth of the Training Set reserved for testing and each of the remaining nine-tenths used in turn for training (Figure 1). Nomogram was constructed based on the results of machine learning models as well as expert discretion.

### 2.7. Assessment of model performance

Four key metrics were used to evaluate the efficacy of the models: area under curve (AUC), sensitivity, specificity, and overall accuracy (ACC). A value of 0.5 < AUC ≤0.7 indicates less accuracy, 0.7 < AUC <0.9 indicates moderate accuracy, and AUC >0.9 indicates high accuracy.

## 3. Results

### 3.1. Laboratory and clinical characteristics

There were 158 ESRD outcome events (40.51%) during the 3-year median follow-up period. The mean age of our participants at biopsy was 51 ± 9.6 years old and men comprised 70% of the participants. The laboratory test results, demographic characteristics, and treatment and renal biopsy results at baseline of the enrolled participants are presented in Table 1. Significant differences were found in sex, sAlb, Hb, sCr, eGFR, diabetic duration, and the use of insulin at the baseline between the ESRD group and the control group (*p* < .05). However, no significant differences were found in the remaining variables (*p* > .05).

**Table 1.** Baseline characteristics of the study population.

	All subjects	ESRD	Control	*p* Value
Number, *n* (%)	390	158 (40.51)	232 (59.49)	
Age (years)	51 ± 9.6	50 ± 8.8	51 ± 10.2	0.247
Sex, men, *n* (%)	273 (70.00)	110 (69.62)	163 (70.26)	0.025
Diabetes duration (months)	97 ± 67	94 ± 65	99 ± 68	0.011
Serum albumin (g/L)	34.34 ± 7.72	30.20 ± 6.65	37.17 ± 7.11	0.002
Hb (g/L)	120 ± 27.15	106 ± 20.52	129 ± 27.05	<0.001
Phosphate (mg/dL)	1.21 ± 0.25	1.27 ± 0.28	1.17 ± 0.23	0.982
Calcium (mmol/L)	2.14 ± 0.17	2.08 ± 0.18	2.19 ± 0.15	0.621
Fasting glucose (mmol/L)	8.32 ± 4.25	8.04 ± 4.15	8.50 ± 4.31	0.338
HbA1c (%)	7.5 ± 1.92	7.2 ± 1.93	7.8 ± 1.89	0.451
HbA1c (mmol/mol)	59 ± 21	56 ± 21.1	61 ± 20.7	
Total cholesterol (mmol/L)	5.16 ± 1.61	5.50 ± 1.74	4.93 ± 1.48	0.086
Triglyceride (mmol/L)	2.19 ± 1.71	2.02 ± 1.48	2.30 ± 1.85	0.251
LDL-C (mmol/L)	2.98 ± 1.28	3.26 ± 1.40	2.79 ± 1.17	0.221
HDL-C (mmol/L)	1.37 ± 0.61	1.43 ± 0.54	1.34 ± 0.66	0.350
eGFR, mL·min^−1^ (1.73 m^2^)^−1^	66.63 ± 34.07	50.89 ± 28.26	77.35 ± 33.58	0.004
sCr (μmol/L)	139 ± 86.32	181 ± 107	110 ± 51.39	0.001
24-hour urine: urinary total protein (g/24 h)	5.28 ± 4.47	6.89 ± 4.61	4.18 ± 4.03	0.799
SBP (mmHg)	146 ± 23.16	149 ± 22.51	144 ± 23.43	0.358
DBP (mmHg)	86 ± 13.12	87 ± 13.10	86 ± 13.15	0.214
BUN (mg/dL)	8.99 ± 5.24	10.45 ± 4.40	7.99 ± 5.54	0.662
Cystatin-C (mg/L)	1.73 ± 0.96	2.09 ± 0.85	1.48 ± 0.95	0.630
UA (μmol/L)	384 ± 86.81	382 ± 76.01	386 ± 93.58	0.919
Use of ACEI or ARB, *n* (%)	306 (78.46)	124 (78.48)	182 (78.45)	0.782
Use of antihypertensive drug, *n* (%)	371 (95.13)	153 (96.84)	220 (94.83)	0.415
Use of insulin, *n* (%)	274 (70.25)	126 (79.75)	148 (63.79)	0.002
Use of hypolipidemic drugs, *n* (%)	242 (62.05)	97 (61.39)	145 (62.50)	0.619
History of smoking, *n* (%)	186 (47.69)	76 (48.10)	110 (47.41)	0.645
Family history of DM, *n* (%)	130 (33.33)	51 (32.28)	79 (34.05)	0.622
Diabetic retinopathy, *n* (%)	175 (44.87)	84 (53.16)	91 (39.22)	0.545
Pathological parameters				
Glomerular class, *n* (%)				
I	19 (4.87)	0 (0)	19 (8.19)	0.772
IIa	85 (21.79)	13 (8.23)	72 (31.03)	
IIb	55 (14.10)	20 (12.66)	35 (15.09)	
III	177 (45.38)	97 (61.39)	80 (34.48)	
IV	54 (13.85)	28 (17.72)	26 (11.21)	
IFTA, *n* (%)				
0	10 (2.56)	0 (0)	10 (4.31)	0.065
1	174 (44.62)	53 (33.54)	121 (52.16)	
2	159 (40.77)	80 (50.63)	79 (34.05)	
3	47 (12.05)	25 (15.82)	22 (9.48)	
Interstitial inflammation, *n* (%)				
0	23 (5.90)	1 (0.63)	22 (9.48)	0.477
1	283 (72.56)	106 (67.09)	177 (76.29)	
2	84 (21.54)	51 (32.28)	33 (14.22)	
Arteriolar hyalinosis, *n* (%)				
0	37 (9.49)	8 (5.06)	29 (12.5)	0.802
1	188 (48.21)	74 (46.84)	114 (49.14)	
2	165 (42.31)	76 (48.10)	89 (38.36)	

Hb: hemoglobin;LDL-C: low-density lipoprotein cholesterol; HDL-C: high-density lipoprotein cholesterol; eGFR: estimated glomerular filtration rate; sCr: serum creatinine; SBP: systolic blood pressure; DBP: diastolic blood pressure; BUN: blood urea nitrogen; UA: uric acid; IFTA: interstitial fibrosis and tubular atrophy.

### 3.2. Pairwise correlations among the patho-clinical parameters

**Figure 2.**
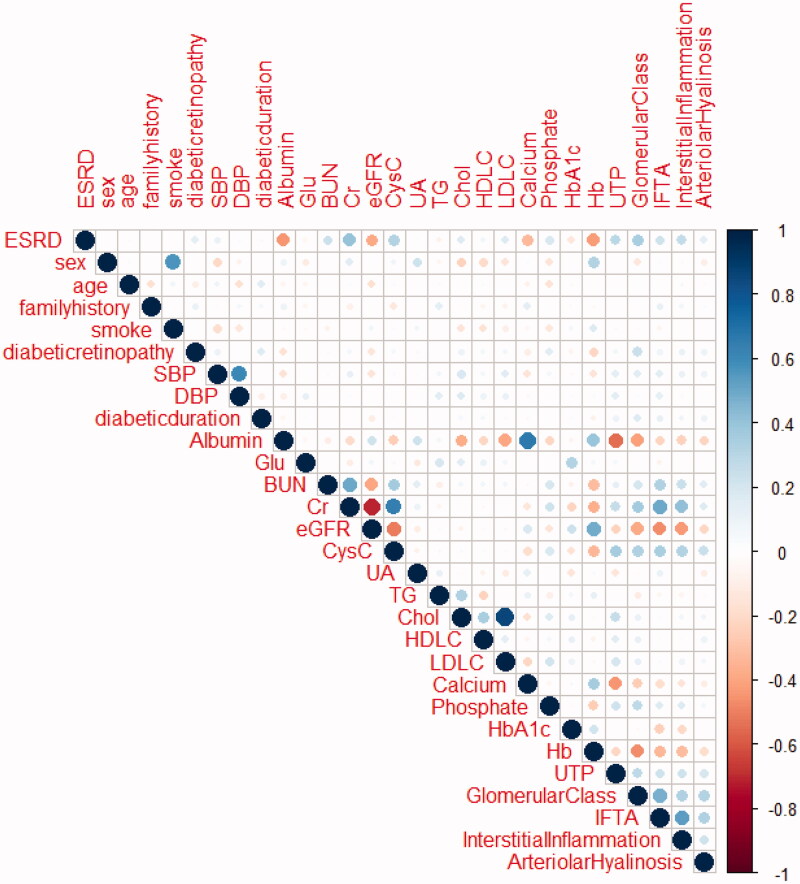
Correlation between variables. The magnitude and direction of the correlation are reflected by the size (larger is stronger) and color (red is negative and blue is posive) of the circles, respectively.

Figure 2 provides an outline of the pairwise correlations between the available patho-clinical parameters for the 390 study participants. The correlation analysis showed significant negative correlations between sAlb, Hb, eGFR, and ESRD, and significant positive correlations between sCr, glomerular class, and ESRD.

### 3.3. Development of the machine learning algorithms

To establish the machine learning models, 292 participants were randomly enrolled in the training set and 98 participants were randomly enrolled in the validation set. Outcomes occurred in 119 participants (40.75%) in the training set and 39 participants (39.80%) in the validation set. Clinical variables including sAlb, fasting glucose, BUN, eGFR, CysC, UA, UTP, HbA1c, TG, Chol, HDLC, LDLC, Hb, phosphate, SBP, DBP, T2DM duration, sex, age, DR, family history, smoking status, pathological parameters of glomerular class, IFTA, interstitial inflammation, arteriolar hyalinosis, C1q, IgG, IgM, IgA, C3, C4 deposition in kidney biopsy samples, and the use of insulin, metformin, diuretics, CCB, ACEI, ARB, statins, and fibrates were used in the training of machine learning classifiers.

**Figure 3.**
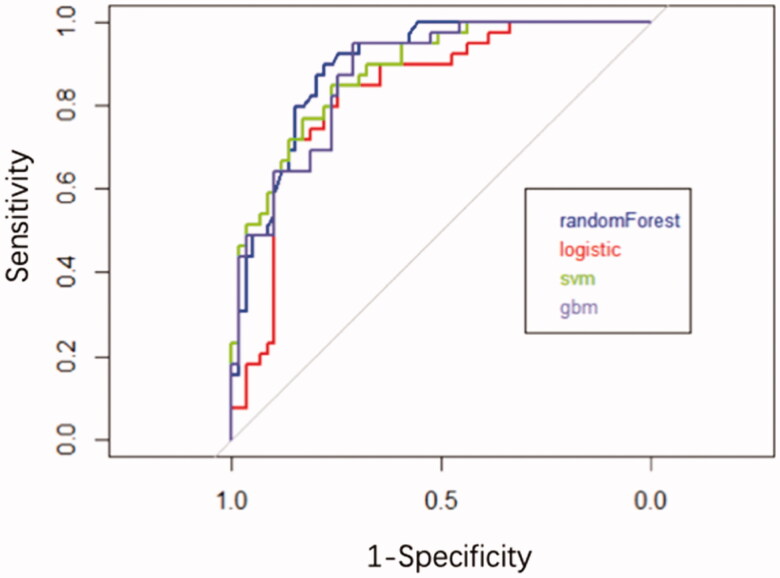
ROC for different machine learning algorithms predicts the results of ESRD in validate data set.

**Figure 4.**
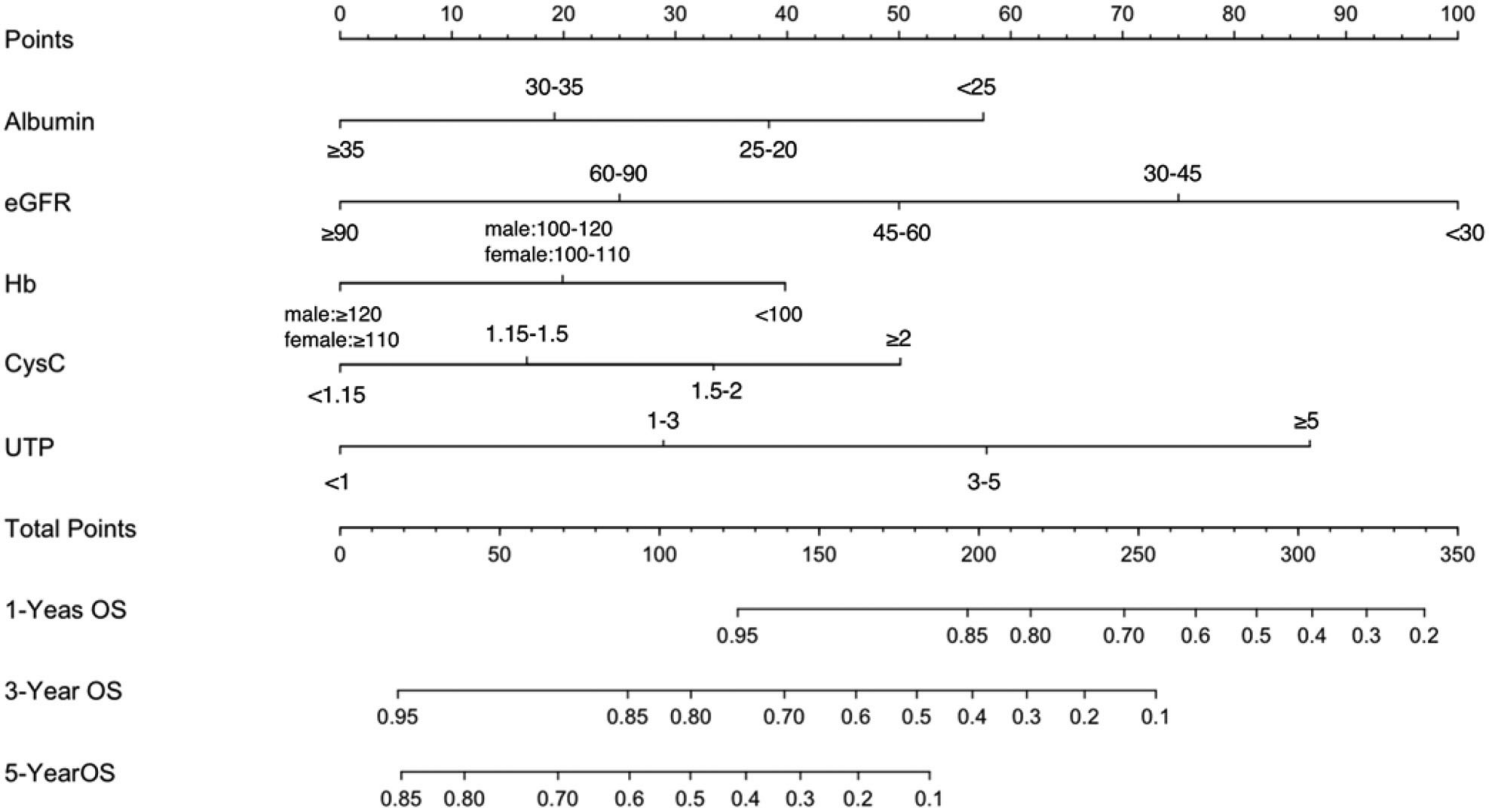
Prognostic nomogram to predict individual renal survival probability in T2DM patients with DKD. The nomogram allows the user to obtain 1-, 3-, and 5-year renal survival corresponding to a patient's combination of variables. Points are assigned for each variable by drawing a straight line upward from the corresponding value to the “Points” line. Then, sum the points received for each variable, and locate the number on the “Total Points” axis. To speculate the patient's renal survival after 1-, 3-, or 5-years, a straight line must be drawn down to the corresponding “1-Year Survival, 3-Year Survival, or 5-Year Survival” probability axis.

Furthermore, we found that the RF algorithm had the highest prediction effect (AUC = 0.90, ACC = 82.65%), following by SVM (AUC = 0.88, ACC = 83.67%), GBM (AUC = 0.88, ACC = 83.67%) and logistic regression (AUC = 0.83, ACC = 79.59%) (Table 2 and Figure 3) in the validation. Therefore, the RF model was chosen to establish the ESRD risk prediction model. According to the MeanDecreaseGINI demonstrating the relative importance of variables (Supplementary Figure 2), the RF algorithm found that the major five factors for the risk prediction model were CysC, sAlb, Hb, eGFR and UTP, followed by BUN, glomerular class, LDL-C, and total cholesterol, while age, fasting glucose, UA, DBP, SBP, HbA1c, HDL-C, phosphate, triglyceride, and diabetic duration showed less importance. To make the model more practical and easier to visualize, we developed a nomogram (C-index = 0.84) using the top five factors (CysC, sAlb, Hb, UTP, and eGFR; Figure 4; Supplementary Tables 1 and 2). Examples of how nomograms help clinicians predict renal prognosis are shown in Supplementary Figure 3.

**Table 2.** Forecast results for invalidation of machine learning algorithms.

Algorithms	AUC	Accuracy (%)	Sensitivity (%)	Specificity (%)
RF	0.90	82.65	83.33	81.58
Logistic regression	0.83	79.59	78.33	81.58
SVM	0.88	83.67	86.67	78.95
GBM	0.88	83.67	95.00	65.79

RF: random forest; SVM: support vector machine; GBM: gradient boosting machine.

## 4. Discussion

After comparing the performance of the above machine learning algorithms in establishing risk prediction models for ESRD in participants with T2DM and DKD, we found that the RF predictive model displayed the highest AUC (0.90) and ACC (82.65%), suggesting that baseline features of participants with DKD can be used to predict renal survival (sensitivity = 83.33% and specificity = 81.58%, respectively). Furthermore, a nomogram based on the top five factors (CysC, sAlb, Hb, UTP, and eGFR) derived from the RF algorithm was developed to predict the possibility of renal survival, which is a simple and practical risk calculator for clinicians.

In the current study, the top five factors in the predictive model partially overlapped with the findings of a previous study [9] (i.e., higher CysC levels, lower eGFR, and higher Log ACR levels). Our previous study had shown that the calculation of eGFR incorporating CysC was better than sCr-based eGFR calculations alone for the early detection of kidney injury [14]. UTP was the fifth-ranked variable in the relative importance of features included in our machine learning model. Conventionally, DKD severity was assessed by measuring urine albumin levels combined with eGFR. Increased urinary albumin excretion had been known as a major risk factor for the DKD progression [15].

In contrast to the findings from the study of Sun et al., we found variables of sAlb and Hb besides CysC levels, eGFR, and Log ACR were critical in predicting ESRD. The differences in the characteristics of the enrolled participants between the two studies may account for such findings. The participants in this study displayed higher sAlb and Hb levels than in the other study. Our previous study had strongly suggested that a lower level of sAlb had association with declining renal function and worse renal prognosis for DKD participants, independent of histopathological and clinical parameters [5]. A machine learning model for predicting long‐term ESRD by Belur Nagaraj et al. [16] also included sAlb as a crucial predictor. A significant inverse correlation of sAlb levels with glomerulopathy and proteinuria might explain the correlation between hypoalbuminemia and the incidence of ESRD. Moreover, the level of sAlb could reflect the degree of oxidative stress and inflammation to some extent. Thus, hypoalbuminemia might accelerate the deterioration of kidney function by inducing endothelial inflammatory injury and oxidative stress [17–19]. The progression of DKD might further lead to reduced energy and protein intake and malnutrition, leading to more severe hypoalbuminemia [20]. Therefore, improving hypoalbuminemia through inhibiting inflammatory state and controlling malnutrition as well as proteinuria might play a crucial role to slow down the progression to ESRD based on our model and previous study.

Furthermore, anemia has been recognized as a sequela of advanced DKD, caused by tubulointerstitial damage [21]. Lower baseline Hb was found to be a risk factor for ESRD in patients with T2DM [22] and in patients with DKD [8]. Anemia induces insufficient oxygen supply in renal tubular cells, low perfusion of capillaries, and damage to energy production, and in involved in the pathogenesis of CKD [23,24]. DKD-related anemia tends to be more severe and develops earlier in comparison with non-DKD-related anemia on the basis of complex mechanisms, such as the inhibitory effects of inflammatory cytokines, poor response to erythropoietin (EPO), and the loss of EPO in urine [25]. Notably, participants who had hypoalbuminemia were also susceptible to being anemic, which could accelerate their kidney damage [26]. Indeed, our group and others had identified hypoxia-mediated pathways as potential therapeutic targets [27]. Since activation of hypoxia-inducible factor (HIF) prevents diabetes-induced tissue hypoxia, proteinuria, and renal tubular interstitial fibrosis by protecting mitochondrial function, HIF improving renal oxygen homeostasis might be a new target for DKD treatment [28]. The inhibition of the sodium/glucose cotransporter 2 was able to suppress HIF-1α and activate HIF-2α and thereby augment erythropoiesis, which can alleviate cellular stress and renal hypoxia, while muting organellar dysfunction, inflammation, and fibrosis^29^.

In the current study, pathological parameters were not incorporated in the Nomogram model and were not as significant as the other predictor factors, which was in contrast to the finding of the study by Sun et al. [9]. Additionally, the addition of glomerular class did not significantly change the predictive performance of the model. That is why RPS scores were not included in our final model. Unfortunately, biopsy-based studies on DKD are limited. The clinical significance of renal biopsy in patients with T2DM with advanced CKD remains controversial. Some studies found that the predicted values of the Kidney Failure Risk Equation and the Diabetic Nephropathy Score (D-score) were not optimal [30]. In the study by Sun et al., the glomerular class of enrolled participants was different from our study (4% vs. 5% of grade I, 7% vs. 22% of grade IIa, 10% vs. 14% of IIb, 63% vs. 45% of grade III and 16% vs. 14% of grade IV, respectively), which may also contribute to the finding discrepancy. Although some pathological changes were found to be associated with dysfunction, further studies are needed to identify which types and grades of pathological lesions and scoring systems are the most appropriate way to predict renal outcomes of DKD.

Interestingly, previous predictive models of the incidence of ESRD in patients with T2DM with or without mild kidney damage identified distinctly different features from our model, which predicted ESRD in patients with T2DM with DKD [2–4]. For example, age and sex were not predictive factors for the incidence of ESRD in participants with T2DM with DKD, which was consistent with the study by Sun et al. [9]. Our previous study indicated that although the rate of rapid progression of kidney disease was relatively low in the youth group, there was no significant difference in the incidence of ESRD among the age groups [31]. Moreover, Wysham et al. showed that age was a significant risk factor related to the risk of DKD and renal-related death for T2DM participants, but not ESRD. Moreover, conflicting findings had been reported in different cohorts and studies had found that the effect of sex was less apparent in DKD than in non-DKD [8]. Taken together, although age and sex were deemed to be crucial factors in the prediction of ESRD in T2DM participants, they might be not as vital in the prediction of ESRD for participants with advanced DKD. Moreover, anti-hypertension, anti-diabetes, and anti-hyperlipidemia medication were selected in the model for participants with T2DM as well, which were not included in the models for participants with advanced DKD. For patients with diabetes, the primary preventative measure is to prevent the occurrence of renal damage, while for patients with diabetes and DKD, delaying the progression and deterioration of renal function to ESRD is paramount throughout every stage of renal damage, since DKD is a chronic disorder with a long duration of disease. The risk factors for DM patients with or without DKD are totally different, even for DKD with different eGFR. DM-care physicians are suggested to strengthen routine and regular monitoring of proteinuria, renal function, serum albumin, and Hb in patients with T2DM and early DKD, pay extra attention to patients with anemia, hypoalbuminemia, proteinuria, and abnormal renal function, and provide early intervention for these patients. Taken together, in contrast with the treatment of participants with early-phase T2DM with or without mild kidney damage, major emphasis should be placed on indicators related to kidney function, nutrition, and anemia for participants with T2DM and advanced DKD to delay the ESRD, rather than age, sex, and control of hypertension and glycemia.

Our study has some notable strengths. First, this is the first study to use machine learning to construct a prediction model for the incidence of ESRD in patients with DKD. Although machine learning is considered to perform well with large databases and big data sets, the RF method is generally recognized for its accuracy and its ability to deal with small sample sizes. In the current study, the RF model showed the highest AUC and ACC, indicating that the model was reliable. Second, the DKD participants of the study were biopsy-confirmed. Third, the pathological parameters of our study were more detailed than those in the model of previous studies. Fourth, although CysC, eGFR, and UTP overlapped with previous models, this is the first study to identify sAlb and Hb as important factors in a predictive model for ESRD. Next, the parameters used in our risk prediction model for ESRD are readily available in the clinic, thus making this model easily applicable in primary care clinical settings. Our predictive model has the potential to recognize participants at high risk for ESRD early in DKD progression, and to provide intensive treatment to those participants to increase the chance of early treatment.

The limitations in our study were as followed. First, participants were registered from a single center and may not represent the Chinese participants as a whole. Further multicenter validation in China and external validation in different ethnic populations is needed. The study sample size was moderate as well. Additionally, some other parameters, such as history of acute kidney injury (AKI), genetic factors, diet, and socioeconomic status, which might also have an effect on survival, were not investigated for all of the participants with DKD at the time of recruitment. Notably, damaged kidney cells from DKD are more susceptible to AKI. Repeated AKI causes maladaptive repair of the diabetic kidney, resulting in the accumulation of irreversible tubulointerstitial fibrosis, which eventually leads to ESRD [32]. Moreover, due to the long enrollment period, changes in treatment plan may have an impact on the prognosis of patients. Regarding the development of the model, the RF algorithm could ignore multicollinearity, so the interpretation of the data may have been affected. Therefore, it is crucial to select the variables in conjunction with the clinical consensus.

Taken together, machine learning algorithms can efficiently predict the incidence of ESRD in patients with DKD. The major predictive factors of ESRD were sAlb, CysC, Hb, eGFR, and UTP. Compared with previous models, the importance of sAlb and Hb levels were highlighted in our model.

## 5. Conclusion

In contrast with the treatment of patients with early-phase T2DM with or without mild kidney damage, major emphasis should be placed on indicators related to kidney function, nutrition, anemia, and proteinuria for patients with T2DM and advanced DKD to delay the onset of ESRD, rather than age, sex, and control of hypertension and glycemia. As nephrologists, we focus more attention on patients with DKD than patients with early-phase DM without DKD and try our best to delay the progression of kidney disease as much as possible. Therefore, clinicians should have different treatment priorities for patients at different phases of T2DM with or without mild kidney damage.

## Informed consent

All patients have given informed consent.

## Consent for publication

Written informed consent for publication was obtained from all participants.

## Data Availability

All codes used during the study are available from the corresponding author by request.

## Data availability statement

The data underlying this article will be shared on reasonable request to the corresponding author.
